# Spiny Projection Neuron Dynamics in Toxin and Transgenic Models of Parkinson’s Disease

**DOI:** 10.3389/fncir.2019.00017

**Published:** 2019-03-15

**Authors:** Yijuan Du, Steven M. Graves

**Affiliations:** Department of Pharmacology, University of Minnesota, Minneapolis, MN, United States

**Keywords:** Parkinson’s disease, striatum, direct pathway, indirect pathway, synaptic plasticity

## Abstract

Parkinson’s disease (PD) is the most common neurodegenerative movement disorder that results from the progressive degeneration of substantia nigra pars compacta (SNc) dopamine (DA) neurons. As a consequence of SNc degeneration, the striatum undergoes DA depletion causing the emergence of motor symptoms such as resting tremor, bradykinesia, postural instability and rigidity. The primary cell type in the striatum is the spiny projection neuron (SPN), which can be divided into two subpopulations, the direct and indirect pathway; the direct pathway innervates the substantia nigra pars reticulata and internal segment of the globus pallidus whereas the indirect pathway innervates the external segment of the globus pallidus. Proper control of movement requires a delicate balance between the two pathways; in PD dysfunction occurs in both cell types and impairments in synaptic plasticity are found in transgenic and toxin rodent models of PD. However, it is difficult to ascertain how the striatum adapts during different stages of PD, particularly during premotor stages. In the natural evolution of PD, patients experience years of degeneration before motor symptoms arise. To model premotor PD, partial lesion rodents and transgenic mice demonstrating progressive nigral degeneration have been and will continue to be assets to the field. Although, rodent models emulating premotor PD are not fully asymptomatic; modest reductions in striatal DA result in cognitive impairments. This mini review article gives a brief summary of SPN dynamics in animal models of PD.

## Introduction

Parkinson’s disease (PD) is the most common neurodegenerative movement disorder with more than six million patients worldwide (GBD 2016 Parkinson’s Disease Collaborators, 2018). PD is largely characterized by the progressive degeneration of substantia nigra pars compacta (SNc) dopamine (DA) neurons that innervate the striatum. The striatum is a critical basal ganglia nucleus involved in initiating and regulating goal-directed movement; in PD, this region undergoes progressive DA depletion leading to the hallmark motor symptoms, i.e., resting tremor, bradykinesia, postural instability, and rigidity. The striatum itself entails a degree of cellular heterogeneity but is primarily dominated by GABAergic spiny projection neurons (SPNs). These SPNs have dendritic arbors heavily populated with spines chiefly consisting of corticostriatal and thalamostriatal circuitry (Bolam et al., 2000; Villalba et al., 2009; Smith et al., 2014). SPNs can be subdivided into two distinct populations based on the DA receptor expression profile and projection patterns. SPNs that express D2 DA receptors project to the external segment of the globus pallidus forming the indirect pathway (iSPNs) and SPNs that express D1 DA receptors project to the substantia nigra pars reticulata and internal segment of the globus pallidus forming the direct pathway (dSPNs). DA effects on each SPN population are distinct; in iSPNs D2 receptor activation engages G_i/o_ proteins, decreases intrinsic excitability and promotes long-term depression (LTD); in contrast D1 receptors in dSPNs couple to G_olf_, increase intrinsic excitability, and promote long-term potentiation (LTP; Surmeier et al., 2007). Animal models of PD have shed light on a number of adaptations that arise in response to DA loss. In this concise review we focus on SPN dynamics in animal models of PD.

## SPN Dynamics and Transgenic Models

Multiple genes have been implicated in PD (SNCA, LRRK2, PRKN, PINK1, and PARK7) and incorporated into transgenic rats and/or mice. A consistent finding across transgenic models of PD is impaired synaptic plasticity. Both dSPN and iSPNs are capable of undergoing forms of cellular learning and memory whereby synapses are weakened (LTD) or strengthened (LTP). In the striatum LTD is selective for corticostriatal circuits (Wu et al., 2015) and induction is achieved post-synaptically with presynaptic expression that is dependent on endocannabinoid signaling. LTD deficits are consistently reported in multiple transgenic models including PINK1 knockout (Kitada et al., 2007; Madeo et al., 2016), LRRK2 knockout (Chou et al., 2014) and A53T synuclein mouse models (Kurz et al., 2010; Tozzi et al., 2012). It is unclear whether the LTD deficit is specific to iSPNs or dSPNs. Some have argued that only iSPNs are capable of undergoing LTD, and thus the deficit only impacts iSPNs (Kreitzer and Malenka, 2007). However, others report LTD in iSPNs and dSPNs (Wang et al., 2006; Shen et al., 2008; Bagetta et al., 2011; Trusel et al., 2015; Wu et al., 2015). The disparity is likely a consequence of intrastriatal electrical stimulation evoking DA release that stimulates D1 receptors, thereby occluding LTD induction. Consistent with this, DA receptor activation uncovers SPN selectivity for LTD (Wu et al., 2015). The impairment of LTD in parkinsonian transgenic mice is a consequence of diminished D2 receptor stimulation resulting in reduced endocannabinoid production but also decreased CB1 receptor expression (Kurz et al., 2010; Madeo et al., 2016); boosting these impairments through CB1 recepetor agonism, increasing DA, or D2 agonism rescues LTD (Kitada et al., 2007; Chou et al., 2014; Madeo et al., 2016). The ability to rescue with D2 agonism may suggest selectivity for iSPNs; however, D2 receptor activation is also involved in dSPN LTD. D2 stimulation on cholinergic interneurons suppresses M1 receptor signaling to dis-inhibit L-type calcium channels allowing for LTD in dSPNs (Wang et al., 2006; Augustin et al., 2018). Nevertheless, based on the classical basal ganglia model it would hypothesize that this impairment is specific to iSPNs. This model posits that the dSPN circuit is the “go” pathway and the iSPN circuit is the “no-go” pathway; dSPN activity thus promotes and iSPN activity suppresses motor activity (Surmeier et al., 2007). In this framework it would be expected that the LTD deficit would be selective for iSPNs thereby biasing striatal output in favor of the indirect “no-go” pathway and motor suppression; consistent with this, deletion of D2 receptors, effectively removing DA-mediated inhibition, in iSPNs impairs motor activity (Lemos et al., 2016; Bello et al., 2017) as does optogenetic stimulation of iSPNs (Kravitz et al., 2010). Further supporting indirect pathway dominance in PD, corticostriatal responses in a lesion model are enhanced and diminished in iSPNs and dSPNs, respectively (Flores-Barrera et al., 2010; Escande et al., 2016), and optogenetic stimulation of dSPNs in parkinsonian mice improves motor function (Kravitz et al., 2010). However, more recent studies demonstrate that coordinated motor activity requires concurrent activation of both iSPN and dSPN populations (Cui et al., 2013; Freeze et al., 2013; Tecuapetla et al., 2014) making it less certain whether the LTD deficits would be pathway specific. Generating transgenic lines with SPN cell-type reporters will be necessary to further dissect circuit-specific changes but it is also important to compare with other PD models independent of genetic mutations.

The generation of genetic models has certainly advanced the understanding of PD; however, these models do not fully emulate the disease state and over 90% of PD cases (late onset) are idiopathic with no known genetic association (Puschmann, 2013). Moreover, transgenic models do not develop neurodegeneration with a few exceptions. In the G2019S LRRK2 model, SNc degeneration (~40%–50%) is reported at 12–16 months of age (Chen et al., 2012) whereas others report no (Yue et al., 2015; Sloan et al., 2016) or very mild (~20%) degeneration (Ramonet et al., 2011) even in 18–21-month-old animals. Reduced tyrosine hydroxylase staining in the SNc is observed in the α-synuclein A53T model at 12 months of age but the magnitude was not quantified (Yamasaki et al., 2016). In contrast, others report no degeneration in A53T mice (Gispert et al., 2003; Kurz et al., 2010). PINK1 animals show no degeneration at 8–9 months (Kitada et al., 2007). Similarly, Parkin deficient animals have no SNc cell loss even at 24 months of age (Goldberg et al., 2003) although animals expressing the Parkin-Q311X mutation show SNc DA neuron loss (40%) at 16 months of age (Lu et al., 2009). These reports support the notion that genetic perturbations increase the vulnerability of SNc DA neurons but are not sufficient for degeneration. SNc degeneration is also not necessary for parkinsonian symptoms. Neuroleptic treatment results in parkinsonism due to D2 receptor antagonism thereby disinhibiting iSPNs and cholinergic interneurons (Kharkwal et al., 2016); chronic neuroleptic treatment even manifests some of the same functional and anatomical changes (Sebel et al., 2017) observed in PD models (Fieblinger et al., 2014). Transgenic models have also not reproduced the changes in SPN dendritic arborization or spine density.

One of the most consistent and reproducible findings in rodent models, non-human primates, and post mortem tissue from patients is a robust loss of spines on SPNs (McNeill et al., 1988; Ingham et al., 1989; Stephens et al., 2005; Zaja-Milatovic et al., 2005; Day et al., 2006; Villalba et al., 2009; Zhang et al., 2013; Anaya-Martínez et al., 2014; Fieblinger et al., 2014; Nishijima et al., 2014; Suárez et al., 2014; Toy et al., 2014; Suarez et al., 2016; Bentea et al., 2017; Gagnon et al., 2017; Gomez et al., 2019); this PD associated anatomical change has not been found (Matikainen-Ankney et al., 2016) or is yet to be reported in transgenic animals with the exception of the Pitx3^−/−^ mouse (Suarez et al., 2018). Both dSPNs and iSPNs of Pitx3^−/−^ mice have decreased spine density. However, Pitx3 is not a gene associated with PD; it is involved in the development of DA neurons. In Pitx3^−/−^ animals, most SNc DA neurons fail to fully differentiate and do not innervate the striatum (Hwang et al., 2003). Thus it is rather a model with neurodevelopmental deficits as opposed to a parkinsonian model of degeneration. The absence of spine pruning in transgenic models of PD may relate to the fact that spine pruning seems to be associated with the severity of DA loss (Villalba et al., 2009; Suárez et al., 2014) and sufficient depletion may not be reached in transgenic mice. The models that reproducibly induce robust degeneration of SNc DA neurons are the toxin models. Toxin models such as the 6-OHDA and MPTP models of PD are far from perfect but comparison with evidence from transgenic models provides a means to determine what is consistent across PD models and thus most likely to be translatable to the human condition.

## SPN Dynamics and Toxin Models

6-OHDA produces a near complete degeneration of DA neurons when injected into the medial forebrain bundle or SNc whereas the same result can be achieved with systemic injection of MPTP. Both toxins produce a rapid and robust loss of DA neurons with overt motor impairment that can be ameliorated by levodopa administration. Consistent with evidence from human post-mortem tissue (McNeill et al., 1988; Stephens et al., 2005; Zaja-Milatovic et al., 2005), both MPTP and 6-OHDA lesioned non-human primates and rodents (Ingham et al., 1989; Day et al., 2006; Villalba et al., 2009; Zhang et al., 2013; Anaya-Martínez et al., 2014; Fieblinger et al., 2014; Nishijima et al., 2014; Suárez et al., 2014; Toy et al., 2014; Ueno et al., 2014; Suarez et al., 2016; Gagnon et al., 2017; Graves and Surmeier, 2019) show reduced SPN dendritic arborization and robust spine pruning in the striatum. However, whether spine pruning is selective for iSPN or dSPNs is debatable. Some report selective spine pruning in iSPNs (Day et al., 2006; Fieblinger et al., 2014; Nishijima et al., 2014); others report decreased spine density in both iSPNs and dSPNs (Villalba et al., 2009; Suárez et al., 2014; Toy et al., 2014; Suarez et al., 2016; Gagnon et al., 2017; Gomez et al., 2019) and it remains to be seen what might account for the disparate findings. One potential variable is the time post-lesion at which investigations are conducted; dSPN spine density is unchanged 30 days post-6-OHDA lesion but decreased 60 days post-lesion (Graves and Surmeier, 2019). The spines of both iSPNs and dSPNs are the sites of glutamatergic axospinous circuitry; this is predominantly corticostriatal circuitry with the remaining subset of axospinous synapses belonging to thalamostriatal circuits. Spine pruning primarily reflects a loss of corticostriatal axospinous synapses (Zhang et al., 2013; Fieblinger et al., 2014; Bentea et al., 2017). Paradoxically, lesioning the motor cortex prevents and even reverses spine loss in 6-OHDA lesioned rats, an effect also achieved by mGluR2/3 antagonism (Garcia et al., 2010). In iSPNs M1 receptors increase dendritic excitability and contribute to spine pruning (Shen et al., 2007). In contrast, M4 signaling in dSPNs promotes LTD at axospinous synapses (Shen et al., 2015) but it is unclear whether this modulates dSPN spine dynamics in PD models. While the transgenic and toxin models disagree when it comes to SPN anatomical changes, consensus is found with synaptic plasticity. Similar to findings in transgenic models, LTD is impaired in both MPTP treated mice (Chen et al., 2008) and monkeys (Quik et al., 2006). LTD is also impaired in iSPNs from reserpinized and 6-OHDA lesioned mice (Kreitzer and Malenka, 2007; Shen et al., 2008; Thiele et al., 2014); rescuing iSPN LTD improves motor impairments (Kreitzer and Malenka, 2007). Based on the above data the LTD impairments in transgenic models are also likely specific to iSPNs. In contrast, LTP is impaired in dSPNs, consistent with a dis-inhibition of cholinergic interneurons and increased M4 stimulation; inhibition of cholinergic interneurons, M4 antagonism, or D1 agonism improves motor performance (Shen et al., 2008; Paille et al., 2010; Thiele et al., 2014; Ztaou et al., 2016). Agonism at D2 receptors also restores impaired LTD in iSPNs similar to the restoration in transgenic mice, whereas D1 receptor agonism restores LTP in dSPNs (Shen et al., 2008); restoring plasticity impairments in both SPN populations also improves motor function (Picconi et al., 2003; Thiele et al., 2014; Trusel et al., 2015).

In addition to spine pruning and impairments of synaptic plasticity, there is a rearrangement of excitatory synapse machinery. MPTP lesioning increases calcium permeable AMPA receptors in iSPNs (VanLeeuwen et al., 2010; Kintz et al., 2013). This is particularly intriguing given the problems with synaptic plasticity in parkinsonian animals; incorporation of calcium permeable AMPA receptors allows for an NMDA-independent form of LTP (Mameli et al., 2011). In this framework iSPNs would have a lower threshold to induce LTP and without sufficient DA to induce LTD this would contribute to indirect pathway dominance.

## SPN Dynamics Preceding Motor Dysfunction

By the time patients with PD are diagnosed and experiencing motor symptoms, >50% of SNc DA neurons have been lost and striatal levels of DA reduced by >60% (Tissingh et al., 1998; Hilker et al., 2005; Cheng et al., 2010). The premotor/prodromal phase is estimated to consist of a 4–6 year period prior to the emergence of motor symptoms and diagnosis (Fearnley and Lees, 1991; Morrish et al., 1998; Marek et al., 2001). During this premotor phase there are likely a number of adaptations occurring within the striatum to compensate for the loss of DA neurons. Human data demonstrate decreased DA reuptake and enhanced DA synthesis in early PD (Nandhagopal et al., 2011); how circuit function is altered and whether alterations are compensatory or pathological is much less clear and an important gap that needs to be addressed. Several groups have made efforts to investigate the premotor phase of PD either through transgenic strategies or partial lesioning of the nigrostriatal system.

PINK1 knockout heterozygous mice are motorically asymptomatic despite having ~40% reduction in extracellular DA (Madeo et al., 2014). These animals exhibit a selective deficit in LTP that is restored by drugs that increase striatal DA including amphetamine, tyramine and selegiline and partially restored by levodopa but not a D1 agonist (Madeo et al., 2014); however, D1 agonsim does restore LTP in homozygous knockout mice (Kitada et al., 2007). In partial 6-OHDA lesioned rats with modest motor impairments, LTP deficits and motor symptoms are also rescued by D1 agonism (Paille et al., 2010). It is unclear why in heterozygous PINK1 knockout LTP is not rescued by D1 stimulation but data support a DA-dependent deficit. Given that LTP in iSPNs utilizes A_2A_ receptors for LTP and D2 receptors promote LTD, it is likely that the LTP deficit is dSPN specific. If this is the case it indicates that the balance between iSPN and dSPN circuit function manifests well before motor symptoms. Consistent with this, in animals that receive a partial 6-OHDA lesion and do not present with motor dysfunction, there is a reduced response in dSPNs to cortical stimulation (Escande et al., 2016). In mice with motor impairment, iSPN corticostriatal responses are increased and dSPN corticostriatal responses further reduced (Escande et al., 2016). Taken together this suggests that dSPN circuits are the first to be impacted by moderate DA depletion and although there are not motor symptoms, animals with partial DA depletion show anxiety- and depression-like behaviors, as well as cognitive impairments (Tadaiesky et al., 2008, 2010; Bonito-Oliva et al., 2014a,b; Tozzi et al., 2016; Loiodice et al., 2018; Ztaou et al., 2018).

The 6-OHDA partial lesion model with 40% DA depletion exhibits impaired spatial object recognition (Ztaou et al., 2018) but with 75% DA depletion, novel object recognition is also impaired (Bonito-Oliva et al., 2014b; Loiodice et al., 2018). Hippocampal circuitry contributes to spatial and recognition memory and with partial lesions, hippocampal DA is reduced and hippocampal LTP impaired (Bonito-Oliva et al., 2014b). Levodopa or D1 agonism, but not D2 agonism rescues the deficits in novel object recognition and hippocampal LTP (Bonito-Oliva et al., 2014b; Loiodice et al., 2018) suggesting that early dysfunction not only occurs in dSPN circuits but are manifesting in D1-dependent systems that extend beyond the striatum. Similar cognitive deficits have also been observed in alpha-synuclein overexpression (Subramaniam et al., 2018). MitoPark mice have progressive degeneration of DA neurons (Ekstrand et al., 2007) with motor impairment beginning at 12 weeks of age (Galter et al., 2010). Preceding the onset of motor symptoms, 8-week-old MitoPark mice also perform poorly in the novel object recognition test and have impaired learning in a spatial task (Li et al., 2013). These cognitive impairments arise at a time when there is a DA deficit but well before the emergence of motor dysfunction.

Impaired striatal function may contribute to impaired spatial learning. MPTP partial lesioned animals are impaired in learning the constant-start version of the water maze task, which involves a cue-dependent navigation strategy within the striatum but performance is unaltered using a variable-start version of the task, which is more dependent on a hippocampal spatial navigation strategy (Da Cunha et al., 2006). The spatial task employed by Li et al. (2013) also used a constant start location paradigm. Therefore, the learning deficit could reflect a striatal dysfunction due to reduced D1 signaling in dSPNs.

## Conclusion and Future Directions

There is no perfect model system to study PD; by examining key findings from the diverse set of preclinical models two themes emerge. First, motorically impaired PD models have impaired synaptic function, particularly in the form of LTP and LTD and these impairments are cell-type specific with LTP being impaired in dSPNs and LTD impaired in iSPNs. Second, dSPNs appear to be the first SPN population to undergo functional alterations in response to moderate DA depletion. At this disease stage subjects are not motorically impaired but are cognitively impaired which may be a combination of dysfunction in dSPN circuits as well as extrastriatal D1 systems. Moving forward it will be important to better investigate changes during premotor phases to understand how the striatum and other DA reliant circuits adapt and deteriorate during disease progression. Moreover, it will be critical to determine what adaptations contribute to motor and non-motor symptoms and which, if any, are fighting to maintain normative function so that treatment strategies can be designed to directly target maladaptive plasticity and support or strengthen the compensatory changes.

## Author Contributions

SG and YD drafted and edited the manuscript. Both authors approved the final manuscript.

## Conflict of Interest Statement

The authors declare that the research was conducted in the absence of any commercial or financial relationships that could be construed as a potential conflict of interest.
